# Inter-kingdom communication and the sympoietic way of life

**DOI:** 10.3389/fcell.2024.1427798

**Published:** 2024-07-12

**Authors:** Scott F. Gilbert

**Affiliations:** ^1^ Department of Biology, Swarthmore College, Swarthmore, PA, United States; ^2^ Evolutionary Phenomics Group, Biotechnology Institute, University of Helsinki, Helsinki, Finland

**Keywords:** symbiosis, sympoiesis, holobiont, development, evolution, cooperation

## Abstract

Organisms are now seen as holobionts, consortia of several species that interact metabolically such that they sustain and scaffold each other’s existence and propagation. Sympoiesis, the development of the symbiotic relationships that form holobionts, is critical for our understanding the origins and maintenance of biodiversity. Rather than being the read-out of a single genome, development has been found to be sympoietic, based on multigenomic interactions between zygote-derived cells and symbiotic microbes. These symbiotic and sympoietic interactions are predicated on the ability of cells from different kingdoms of life (e.g., bacteria and animals) to communicate with one another and to have their chemical signals interpreted in a manner that facilitates development. Sympoiesis, the creation of an entity by the interactions of other entities, is commonly seen in embryogenesis (e.g., the creation of lenses and retinas through the interaction of brain and epidermal compartments). In holobiont sympoiesis, interactions between partners of different domains of life interact to form organs and biofilms, wherein each of these domains acts as the environment for the other. If evolution is forged by changes in development, and if symbionts are routinely involved in our development, then changes in sympoiesis can constitute an important factor in evolution.

## 1 Introduction: our holobiont heritage

Evolutionary developmental biology is charged with describing and appreciating the mechanisms by which selectable variation is generated, maintained, and propagated. It is the science directly concerned with the origins of the planet’s biodiversity. But to do this, it must first have an understanding of what it is that changes. That is to say, if evo-devo is to explain organismal diversity, it must know what an organism actually is. The concept of organism has evolved in a new direction during the past 20 years. Keller (2002) characterized the 20th century as “the century of the gene,” but the twenty-first century may become identified as the century of the holobiont relationship. We have become cognizant that multicellular organisms are not (and have never been) biological individuals (Gilbert et al., 2012; McFall-Ngai, 2024). Rather than being the read-out or epiphenomena of genes, our anatomy, physiology, immunity, development, mental health, and evolution are now seen to be performed in concert with other symbiotic organisms. I will argue that appreciating that developing organisms are holobionts (the organism as consortium of symbionts) has important consequences for modeling development, evolution, and the history of life.

“Organisms are holobionts, and life is sympoietic,” is a statement that could not have been made from 20th century biology. The term “holobiont” refers to the scientific conclusion that organisms are integrated consortia of a host organism plus numerous species of other symbiotic organisms (Zilber-Rosenberg and Rosenberg, 2008; Theis et al., 2016)^1^. In the adult human body, microbes account for approximately half of our cells (Sender et al., 2016). Moreover, these bacteria, fungi, protists, and archaea are not just travelling in our body and sharing our food. They are critical for our healthy physiology, development, and immunity (Gilbert et al., 2012; McFall-Ngai et al., 2013). Cows, for example, may be herbivores; but there are no genes in their bovine nuclei that encode grass-digesting enzymes. These cellulose-digesting enzymes come from the set of microbes living within the rumen of the cattle’s guts (Moraïs and Mizrahi, 2019a). In coral, most of the animal’s carbon resources are derived from the photosynthetic reactions of its algal symbionts (Muscatine et al., 1984). Symbiotic fungi extend the roots of plants, allowing them to get water and nutrients more efficiently. These fungal extensions may have been important for permitting plants to adapt to land (Pirozynski and Malloch, 1975). Multicellular organisms exist as multi-species consortia.

Moreover, even “unicellular organisms” are not truly unicellular. Rather, eukaryotic protists (amoebae and diatoms, for instance) are holobionts and are associated with microbial symbionts (Vincent et al., 2018; Kanso et al., 2021; Colp and Archibald, 2021). The protist *Mixotricha paradoxa* is largely responsible for synthesizing the lignin-digesting enzymes that permit certain termites to metabolize wood. Only, *Mixotricha* is not a single cell, but a protist whose eukaryotic cell is part of a consortium that includes thousands of bacteria, comprising at least four different species (Margulis and Sagan, 2001). With few (if any) exceptions, animals and plants are holobionts, federated partnerships of numerous species functioning together to generate a healthy organism^2^ (Zilber-Rosenberg et al., 2008; Theis et al., 2016; Roughgarden et al., 2017). An organism is not a monoculture of genetically identical cells.

We now understand, that metabolism is a communal enterprise (Dupré and O'Malley, 2009; Kelty, 2019). In holobionts, the set of symbiotic organisms (including the “host”) participate in each other’s metabolisms. In the mealy bug *Planococcus*, the production of the amino acid phenylalanine begins with enzymes encoded by the bug’s symbionts, *Tremblaya* bacteria. The metabolites generated by these enzymes then travel into another bacterial species, *Moranella*, which are symbionts within the *Tremblaya* bacteria. The newly made metabolites from *Moranella* then return to the *Tremblaya* bacteria, wherein they are converted into a product that can be metabolized into phenylalanine by enzymes encoded by the *Planococcus* genome (McCutcheon and von Dohlen, 2011).

In mammals, bacterial products are sent through the circulation of the body, where they sustain digestion, blood circulation, and other physiological functions (McFall-Ngai et al., 2013; Kelty, 2019). In this way, murine gut microbes convert dietary tryptophan into indole, which circulates through the blood and enters the hippocampus. Here, it activates the aryl hydrocarbon receptors of the neural stem cells, converting the receptor into a functional transcription factor that activates the genes responsible for generating neurons. Since these neurons are thought to be critical for memory and learning, the “gut microbes are an evolving, prokaryotic component of the meta-organismal self” (Wei et al., 2021, p. 2). The gut neurons of the esophagus are also beneficiaries of bacterial products. These neurons create the peristaltic waves of muscle contractions that transport food from the throat to the stomach. However, at birth, these neurons are immature. The bacteria produce products (probably short-chain fatty acids) that induce the intestinal cells to synthesize and secrete the hormone serotonin. The serotonin promotes the maturation of the immature neurons and allows efficient peristalsis (De Vadder et al., 2018; Liu et al., 2024).

In 2013, Smith and colleagues coined the term “co-metabolism” to indicate that foods are being metabolized by both host enzymes and microbial enzymes, and that the products of one are often substrates for the other. We have an entangled metabolism. We are not merely organisms; we are biomes, collections of ecosystems (Gilbert, 2019; Suarez and Stencel, 2020). Our development, then, includes both embryogenesis and ecological succession (Blaser and Kirschner, 2007; Gilbert, 2023).

## 2 Transmission of symbionts

In most all organisms, symbionts are needed to establish or complete normal development. Therefore, the transmission of symbionts from one generation to the next is a crucial feature of their life cycle. Animals receive their first set of microbes in many ways (Funkhauser and Bordenstein, 2013; Theis et al., 2016; Roughgarden et al., 2017). Those organisms using intra-organismal transmission transfer symbionts directly from parent to offspring. Other organisms use intimate neighborhood transmission, wherein a parent provides symbionts as resources at birth or shortly thereafter. In addition, some species utilize a horizontal transmission of symbionts, where the offspring are not given symbionts but inherit a means by which they can select them from the environment. Most organisms probably use combinations of these mechanisms over their lifespan (see Bright and Bugheresi, 2010).

### 2.1 Intra-organismal transmission

Those organisms using the intra-organismal transmission methods can impart symbionts to their offspring through: 1) vegetative reproduction, 2) oocytes, or 3) embryos. In hydra, budding allows the generative fragments to contain both the nucleated cells and bacterial symbionts (Minten-Lange and Fraune, 2021). In *Drosophila*, for instance, the *Wolbachia* that provide the *Drosophila* fly with immunity against viruses (Hedges et al., 2008; Teixeira et al., 2008) become concentrated in the posterior pole of the embryo, the region that will become the oocytes and the trophocytes (“nurse cells”) that feed the oocytes. These bacteria are transported (along the same cytoskeletal pathways as mitochondria, mRNA, and ribosomes) from these gonadal cells into the oocyte, from which they will spread throughout the body (Ferree et al., 2005; Newton et al., 2015). In pea aphids, the obligate symbiont *Buchnera* is transmitted by exocytosis from the mother’s gut into the posterior region of the developing embryo (Koga et al., 2012).

Since bacteria are transmitted to the next-generation only by eggs, several *Wolbachia* species can increase female fecundity (and hence its own propagation) by causing the oocyte precursor cells to undergo extra divisions (Fast et al., 2011; Guo et al., 2020). Moreover, in some insects, *Wolbachia* can even transform male embryos into female embryos, thus continuing its propagation (Ote and Yamamoto, 2020).

The poster-organisms for holobionts, the lichens, emerge from a photosynthetic organism (the “photobiont,” an algae or cyanobacteria) engaging in mutualistic symbiosis with a fungus (often called the “mycobiont” partner). The photosynthesis of the photobionts produce carbohydrates that benefit the fungus, while the photobiont benefits from the fungal hyphae that anchor them to and accumulate moisture and nutrients from the environment. When undergoing asexual reproduction, pieces of existing lichen break off, and all the species travel together. In some species, small groups of alge (and perhaps yeasts) are enveloped in a fungal basket (a soredium) and dispersed. Once the soredium settles, a new lichen can form. Other asexual lichens elongate fungal outgrowths (isidia) that are dispersed to provide a fungus that readily recruits photobionts. Sexual reproduction also occurs in the fungi of most lichen species to form haploid fungal spores. When these disperse and germinate, they initiate a new fungal colony that can recruit photobionts. Remarkably, it is not difficult for fungi and algae to find each other (Hom and Murray, 2014), and this may be an important feature in expanding the domain of living organisms.

### 2.2 Intimate neighborhood transmission

#### 2.2.1 Coprophagy

One of the most efficient ways of transmitting symbionts from parent to offspring is by coprophagy--the ingestion of feces (Linaje et al., 2004; Kovacs et al., 2006). Many insects lay eggs in or on their feces, which the larval offspring consume upon hatching (Blum et al., 2013; Jahnes et al., 2021). This is especially important for herbivores, since the feces that the larvae eat are comprised mostly of cellulose. Most insects do not have the genes that synthesize cellulose-digesting enzymes, and such fecal pellets lack most essential amino acids. These enzymes and amino acids are produced by the microbes. In many dung beetles, the mother rolls mammalian dung into a ball, buries the ball, and lays an early embryo atop it. But between the dung ball and the embryo she excretes a *pedestal*, a pellet of her own feces that contains the microbial symbionts that can digest the plant-containing dung dropped by the herbivores (Ledón-Rettig et al., 2018; Rohner and Moczek, 2024).

The squash bug *Anasa tristis* requires the bacterial symbiont *Caballeronia* for normal growth and development (Acavedo et al., 2024). While the squash bug is born without these symbionts, it acquires them as immature newly hatched nymphs. The nymphs can accurately smell the presence of *Caballeronia*-containing feces left by adult squash beetles. Moreover, using fluorescently-tagged microbes, it was shown that this preference was specifically for the *Caballeronia* bacteria and not for a species-specific component of the feces.

Coprophagy can take many forms. In some species of termites, workers feed newly hatched juveniles the feces of adults (a process that has been given the wonderful name, proctodeal trophallaxis) (Brune and Dietrich, 2015). Koalas use a special adaptation of coprophagy (Osawa et al., 1993) wherein the mother feeds her infant a mixture of milk and feces, which she smears on their faces. The microbes are able to colonize the gut, permitting the newly weaned joey to digest eucalyptus leaves. Some species of caecilians, a legless amphibian group, transmit their symbiotic bacteria through their skin. The females tend their newborns, and the newborns then eat their mother’s particularly fatty skin. The young amphibians thereby acquire nutrition and a set of symbiotic microbes on their skin and in their gut (Kouete et al., 2023).

#### 2.2.2 Vaginal delivery

In humans and most other mammals, a starter set of microbes colonizes our bodies as the fetus passes through the birth canal. During the third trimester of pregnancy, particular species of microbes are selected in the female reproductive tract and anus. Many of these are spore-forming bacteria that can withstand the different conditions they will encounter (Browne et al., 2016), and this property facilitates transmission. These bacteria appear to help a pregnant woman adapt to the physiological stresses of carrying a fetus and also establish the conditions that enable other bacteria to find niches in the intestines (Koren et al., 2012). In humans, there is a remarkable “crosstalk between our human “first genome” and microbial “second genome.” Wacklin et al., 2011; Zhernakova et al., 2024), and expression of the *ABO* (blood group) and *FUT2* (secretor) genes allows the gut epithelium to select for different bacterial groups (Rausch et al., 2017; Rühlemann et al., 2021).

In addition, mammalian milk provides microbes and a microbe-promoting diet from their mothers. Milk provides an additional route for microbes to travel from parent to offspring (Jin et al., 2011; Jost et al., 2013; Addis et al., 2016). Human milk contains about 10^5^ bacteria per ml, and it comprises hundreds of species. The DNA of bacterial strains isolated from mother’s milk appears very similar to the DNA found in the offspring’s gut (Milani et al., 2015). In addition to providing bacteria, mother’s milk contains complex oligosaccharides that support the growth of the major group of these bacteria, *Bifidobacterium*, but which are not digestible by the infant or by other bacteria (Sela et al., 2011). Bifidobacterium contains genes encoding the enzymes that digest these oligosaccharides (Garrido et al., 2016 (Zivkovic et al., 2011; de Muinck and Trosvik, 2018);^3^. Suggesting that symbiotic bacteria and their human host co-evolved. The gut microbiome will continue to expand as the baby becomes part of a complex environment (Kort et al., 2014; Milani et al., 2017; Stewart et al., 2018).

### 2.3 Horizontal acquisition

Last, symbionts can be acquired from the environment. This probably happens in most animals, but it is critical for those animals whose development requires these organisms that are not supplied by their parents. These animals inherit the ability to recognize and respond to particular environmental microbes, which become affordances^4^ for normal development. This “horizontal” or “environmental” recruitment of symbionts also allows juvenile animals to use microbes from the environment to create diverse phenotypes that depend on the symbiont.

The Hawaiian bobtail squid, *Euprymna scolopes*, is the best studied organism for the horizontal transmission of symbionts. A 2-inch long swimmer of shallow Hawaiian waters off Hawaii, this nocturnal squid preys on shrimp. However, predatory fish can readily see the squid if the moon casts the squid’s shadow on the sea floor. *Euprymna scolopes*, has solved this problem by developing a ventral light that can shine on the seafloor and hide its shadow from potential predators. Making this light organ requires the help of the symbiotic bacterium *Vibrio fischeri*. Indeed, it is the bacteria that glow. It has to collect these bacteria, and the bacteria have to build the light organ. Other species of bacteria in the sea water attempt to stick to the squid, but the squid repels these bacteria species with toxic mucus, acid, and nitric oxide (Troll et al., 2010; Wang et al., 2010; Schwartzman et al., 2019). *Vibrio fischeri* survive these chemicals and induce gene expression changes in the squid epithelial cells, allowing the *V. fischeri* to aggregate (Altura et al., 2013).

Once aggregated, the bacteria migrate inward through a gradient of chitobiose made by the epithelial cells (Mandel et al., 2012; Kremer et al., 2013). Inside the crypts, the bacteria’s bioluminescence is stimulated through a quorum-sensing feedback loop. At critical densities, the bacteria produce two molecules that induce transcription of their own *lux* genes, which are the genes responsible for luminescence (Visick et al., 2000; Millikan and Ruby, 2001). Thus, the light organ is a product of inter-kingdom communication.

### 2.4 Community ecology as part of holobiont development

While embryonic development and organismal succession are usually studied in the separate fields of developmental biology and ecology, respectively, they are united in the holobiont. If we take seriously the idea that organisms are both individuals and collections of ecological communities, then we have to realize that animals are simultaneously the products of embryological development and ecological succession (Gilbert, 2023). Indeed, Skillings (2016) maintains that “most holobionts share more affinities with communities than they do with organisms.”

Ley and colleagues (2007, p. 3) note, “When a new human being emerges from its mother, a new island pops up in microbial space.” And if we are islands popping up in microbial space, then the colonization and succession patterns of island biogeography prevail. We should therefore look at mammalian development in terms of dispersal, local diversification, environmental selection, and ecological drift (Costello et al., 2012). Our various microbiomes have ecological succession (Gonzales et al., 2011; Bordenstein and Theis, 2015; Ratsika et al., 2022), resulting from a combobulation of several factors (Milani et al., 2017), including the bacteria available for colonization (Wampach et al., 2018; Shao et al., 2019), the diet that preferentially enables the proliferation of certain microbes (Carmody et al., 2015; Rothschild et al., 2018), and the genotype of the organisms that provide the environment for the microbes^5^ (Goodrich et al., 2014; Brooks et al., 2016; Kurilshikov et al., 2021; Zhernakova et al., 2024).

Breast-fed and formula-fed macaque monkeys, for instance, generate different communities of gut microbiota, and the microbiota of such breast-fed monkeys are more capable of producing the lymphocytes responsible for eliminating opportunistic pathogens (Ardeshir et al., 2014). Here, diet produces two alternative equilibrium states with different capacities. In other cases, different populations of microbes act to perform similar functions (Doolittle and Inkpen, 2018). Different herds of cattle have different symbiotic populations of microbes in their rumen. Although each of these microbial communities allow sympoiesis and nutritional symbiosis, they can have different phenotypes. Some of these communities, for instance, produce higher level of methane gas than do others (Moraïs and Mizrahi, 2019). Some microbes are generalists and will colonize numerous species, whereas other microbes are specialists that recognize species-specific genetic markers on particular hosts (Theis et al., 2016; Lim and Bordenstein, 2020).

## 3 Sympoiesis

### 3.1 Generalized sympoiesis

The example of *Euprymna/Vibrio* brings us to sympoiesis, the mutualistic partnerships to generate a phenotype, a “making-with” process whereby development occurs as a team activity. Indeed, as important as symbiosis is to the adult organism, such symbioses are not just between mutually consenting adults. Symbiosis occurs during development to *generate* the adult. Sympoiesis refers to the observation that multicellular eukaryotic organisms use symbiotic microbes to co-construct organs. It is a critique of autopoiesis, the idea that organisms are self-generating (Dempster, 1998; Haraway, 2016; Clarke and Gilbert, 2022).

Sympoiesis should be familiar to embryologists. When the bulge from the brain meets the surface ectoderm of the head, it tells the ectoderm that it will become lens. Reciprocally, as the lens begins to form, it tell the brain bulge cells that they will become retina. In this way, the eye will be formed. The lens and retina co-constructed each other. Neither existed previously. The dozen cell types of the kidney are made by the interactions of the ureteric bud and the mesonephrogenic mesoderm, neither of which would have made any structure on its own (see Barresi and Gilbert, 2023). Similarly, *Vibrio* bacteria will activate the *Euprymna* squid genes needed to form a functional light organ (McFall-Ngai, 2021), and the squid will tell the bacterial genes to produce light (Dunn et al., 2006). The functional light organ did not exist before the bacteria and the squid cells made it together.

Animal (and plant) development is predicated on such sympoieses. The nerves that promote peristalsis and hearing, the lymphocytes that constitute the immune system, the intestinal capillaries that take nutrients to the body, and even portions of the mammalian brain are generated or matured by microbial symbionts (Steppenbach et al., 2002; see Gilbert and Epel, 2015). Indeed, the cow rumen is constructed in response to signals from the bacteria whose progeny will dwell within it (Sander et al., 1959; Baldwin and Conner, 2017). *Wolbachia* bacteria are responsible for the proper orientation of the second mitotic division of the nematode *Brugia malayi* (Landman et al., 2014), and they are needed for the formation of ovaries in the *Asobara* wasp (Dedeine et al., 2001). Almost all animals are co-created through interactions between their zygotically derived cells and their environmentally derived microbes^3^.

Gut microbes were crucial for orchestrating gut formation in mammals. The mammalian gut becomes colonized by bacteria as the fetuses pass through the mother’s birth canal. Stappenbeck and colleagues (2002) demonstrated that in the absence of these normal intestinal bacteria, the capillaries lining the villi of the small intestine fail to develop complete vascular networks. Microarray analyses of mouse intestinal cells show that the normally occurring gut bacteria upregulates the transcription of numerous mouse intestinal genes, including those encoding colipase (which is important in nutrient absorption), angiogenin-4 (which promotes the formation of blood vessels) and Sprr2a (a small, proline-rich protein that is thought to fortify matrices that line the intestine). In other words, products of the bacterial cells can induce gene expression in the mammalian intestinal cells (Hooper and Gordon, 2001). Certain gut microbes, mainly strains of *Bacteroides*, are critical for activating the transcription of the gene encoding angiogenin-4 in the Paneth cells of the intestine.

The mRNA for angiogenin-4 is translated into the angiogenin-4 protein, which is then secreted and induces the mesodermal cells lining the intestine to organize into capillaries. But *Bacteroides* bacteria and angiogenin-4 have other properties, as well. First, angiogenin-4 acts through Wnt and Notch to expand the population of Lgr5^+^ intestinal stem cells. At higher concentrations it causes apoptosis of these cells, thereby becoming a regulator of intestinal homeostasis (Abo et al., 2023). Second, Ang4 is toxic to *Listeria*, *Candida* and *Salmonella*, potential competitors of *Bacteroides.* Administration of Ang4 not only increased beneficial bacteria such as *Lactobacillus, Akkermansia*, *Dubosiella*, and *Adlercreutzia*, but also decreased certain pathogenic bacteria, including *Alistipes* and *Enterohabdus*, indicating that angiogenin-4 “regulates the shape of gut microbiota composition” (Sultana et al., 2022). Angiogenin-4 is also toxic to *Bacteroides thetaiotamicron*, preventing it from dominating the bacterial population. Third, *Bacteroides thetaiotaomicron* secretes a DNase to form bile-dependent biofilms. Just as bacteria induce gut cells, the gut induces new properties in the microbes. “Physiological concentrations of bile extract induce the formation of biofilm in almost all tested *B. thetaiotaomicron* strains” (Béchon et al., 2022). Therefore, the microbes help establish and regulate the gut microenvironment. Moreover, it is the holobiont community--the zygotic-derived cells and the bacteria--that work together to protect it from invasions of other external bacteria (Chiu et al., 2017).

Zebrafish guts also have a vast assortment of microbes (see Jemielita et al., 2014), and these microbial symbionts activate the Wnt paracrine signaling pathway to initiate cell division in the intestinal stem cells (Rawls et al., 2004). Without this bacterially induced stem cell division, the zebrafish have thinner intestines, with a deficiency of enteroendocrine and goblet cells (Bates et al., 2006). The region of the gut that forms the zebrafish pancreas and its insulin-secreting beta-cells is also induced by bacteria. In zebrafish, the proliferation of insulin-secreting pancreatic beta cells depends on a protein generated by a relatively rare microbe, *Aeromonas* (Hill et al., 2016; 2022). This bacterial species contains a gene*, BefA*, whose secreted protein product stimulates the proliferation of beta cells in the zebrafish from their fetal levels to their adult levels. Zebrafish lacking *Aeromonas* (or whose *Aeromonas* bacteria lack the *BefA* gene) have a small number of beta cells and a diabetes-like syndrome. This number can be expanded to normal levels, however, by supplying the fish with the protein synthesized by the wild-type *BefA* gene. Moreover, providing additional BefA protein to diabetic mice alleviated their diabetic symptoms by increasing the number of beta cells (Wang et al., 2022).

Indeed, insulin signaling may be a multi-species effort throughout the animal kingdom. *Drosophila* larvae eating the rotten fruit upon which their egg was laid acquire about 20 species of bacteria and yeasts in their guts. Some of these microbes produce compounds that stimulate insulin synthesis. The insulin regulates the speed of developmental as well as the body size, energy metabolism, and intestinal stem cell activity of the larvae (Douglas, 2011; Shin et al., 2011; Storelli et al., 2011).

### 3.2 The sympoiesis of the rumen ecosystem

One of the most important of the sympoietic events has been the generation of the rumen in cattle. The cow’s rumen is the example *par* excellence of an ecosystem residing within an organism. As a recent review (Mizrahi and Jami, 2021) succinctly notes, “Ruminants, more than any other mammalian group, also represents the epitome of mammalian-microbe symbiosis, as they rely completely on microbial fermentation to sustain their lives.” A cow is an herbivore that is dependent upon digesting grass. But it cannot digest grass alone. The genome of the domestic cattle, *Bos taurus,* makes no enzymes capable of digesting cellulose or other plant wall compounds. The digestion of plant materials in both these organisms is accomplished by symbiotic microbes that resides within their guts. Cattle can only survive as holobionts. They are rumnants, as they have a specialized region of their gut, the rumen, that houses these symbiotic microbes. A rumen can be simultaneously viewed as an organ, a complex ecosystem, and “a large anaerobic fermentation chamber where plant-degrading rumen microbiota (bacteria, protozoa, archaea, and fungi) ferment otherwise non-digestible plant-based foodstuffs into primarily the volatile fatty acids acetate, propionate, and butyrate” (Daniels and Yohe, 2014).

The microbial composition of the rumen changes in a way that resembles ecological succession. The first colonizers of the rumen (i.e., the bacteria acquired in the passage through the birth canal) are aerobic bacteria. However, these bacteria utilize the oxygen in the ruminal region over the next 2 days, so this volume becomes populated by anaerobic bacteria that can survive without oxygen (Jami et al., 2013). The microbial ecosystem of the rumen from then on depends on the starter set of microbes it receives from its mother, the microbes it receives from its environment, the diet of the animal, and the genome of the animal. Different breeds of cow select for different microbes, and different locations allow different microbes to colonize the gut. As Taxis and colleagues (2015) have shown, “the players may change but the game remains.” The functions are what’s important, not the species that perform them. As Doolittle and Booth (2017) have noted, “it’s the song not the singer” that’s critical.

Still, there does exist a core community of microbial species, some of which are found in 100% of cows surveyed. These are the “keystones” of rumen ecosystem. Two of them are *Ruminococcus flavifaciens* and *Fibrobacter succinogenes,* bacteria capable of digesting cellulose into soluble sugars. Another keystone genus, *Prevotella,* appears important for converting products to short-chain fatty acids (SCFA) such as propionate and butyrate (Wallace et al., 2019; Furman et al., 2020). So in addition to “It is the Song Not the Singer,” there is also “Nobody Does It Better.”^6^

But in addition to this nutritional *symbiosis* in the adult cow, there is also *sympoiesis* between the microbes and the bovine cells to *generate* the rumen. The calf is not born with an anatomically or physiologically mature rumen. The gut gets colonized by bacteria, but in the distal esophagus and proximal stomach, these bacteria are not given food to metabolize (Warner, 1956; Giesecke et al., 1979; Daniels and Yohe, 2014; Malmuthuge et al., 2019). Rather, the newborn calf is fed milk, and this milk passes through a duct, the esophageal groove, that bypasses the rumen. When the calf is weaned and feeds on grass or grain, the groove disappears, the plant material enters the proximal stomach, and the bacteria digest and ferment these plant cell wall carbohydrates into short chained fatty acids. Butyric acid induces new gene expression patterns in this region of the gut, and the immature rumen is converted into a functional rumen (Baldwin and Conner, 2017; Liu et al., 2024). Calves that retain a milk-only diet do not mature their rumen, and adding butyric acid to an unweaned calf’s diet will cause the rumen to develop prematurely (Sander et al., 1959; Baldwin and Conner, 2017). In other words, as the bacteria proliferate by metabolizing the grasses and grains, they produce the short chain fatty acids that cause the rumen to differentiate and grow.

It appears that *Prevotella*, *Bacteroides*, and *Ruminococcus* bacteria work together to initiate new transcription in the rumen (Malmuthuge et al., 2019). They create SCFA from cellulose, and the SCFA activates the genes for numerous transcription factors that promote the growth and differentiation of rumen tissue become activated. This new activation of gene expression appears to be accomplished by acetylating specific nucleosomes. When butyrate is given to cultured rumen cells in the laboratory, the levels of nucleosome modification correspond to the levels of butyrate, and there is a correlation between the levels of butyrate and the developmental maturity of the rumen (Deng et al., 2023; Kang et al., 2023). Thus, the bacteria help construct the rumen, which will house them and allow the survival of the host organism. Here we see both symbiosis in the adult and sympoiesis in the juvenile (Chiu and Gilbert, 2020). What is critical is understanding that the cow is a holobiont that develops both as an organism (the product of zygotic cells) and as the ecosystem (of the acquired cells).

### 3.3 Sympoiesis of cognitive systems

The development of mammalian immune and nervous systems, the major cognitive interfaces that allow us to interact with our environments (Tauber, 2013) develop sympoietically. Germ-free mice have serious neurological and immunological deficiencies. Symbiotic gut microbes are required for generating the B-cells and T-cells of the gut-associated lymphoid tissue (Rhee et al., 2004), and the immune systems of such mice have fewer lymphocytes, less active intestinal macrophages, lower cytokine production, and lower titers of serum immunoglobulin (Dobber et al., 1992; Kieper et al., 2005; Mazmanian et al., 2005; Ratsika et al., 2022). Therefore, as mentioned in our earlier discussion of angiogenin-4, immunity is a holobiont property, made through sympoiesis. It is not merely a function of the host (Gilbert and Tauber, 2016; Pradeu, 2019; Schneider, 2021)^7^.

The mammalian brain also develops sympoietically (Cryan et al., 2019; Morais et al., 2021; Nagpal and Cryan, 2021). Compared to conventionally bred mice, germ-free mice have higher titers of serotonin, while maintaining lower levels of the mRNAs for transcription factor Egr1 and the paracrine factor BDNF in the relevant portions of their brains (Diaz et al., 2011; Clarke and Gilbert, 2022). These changes correlate with behavioral differences between the germ-free and conventionally raised mice. Diaz Heijtz and colleagues (2011, p 3047) concluded that “during evolution, the colonization of gut microbiota has become integrated into the programming of brain development, affecting motor control and anxiety-like behavior.”

Although mammalian embryos may develop within an aseptic amnion (Kennedy et al., 2023), about 30% of the metabolome coursing through the maternal bloodstream of a pregnant mouse emerges directly or indirectly from symbiotic microbes (Nicholson et al., 2012; McFall-Ngai et al., 2013). Therefore, microbes in the maternal gut can create compounds that circulate throughout the body, enter into the fetal circulation, and affect the developing fetus. When the gut microbes of a pregnant mouse digest and ferment plant fibers, they produce short-chain fatty acids (such as those seen to direct the development of the cattle rumen) that enter the maternal bloodstream and enter the organs of her developing embryos. In the embryo, these bacterial products activate particular genes in the pancreas, nervous system, and intestines, generating proteins that mature the sympathetic neurons and provide life-long obesity-resisting metabolic phenotypes to the offspring (Kimura et al., 2020). Other soluble digestion products made by the maternal gut microbiome (e.g., hippurate and trimethyl-5-aminovalerate) stimulate the maturation of auditory neurons in the fetal mouse brain (Vuong et al., 2020). If these latter metabolites were not generated by the pregnant maternal microbiome, her adult progeny have hearing deficiencies.

If the microbes of mammalian guts appear to be crucial for stimulating “basic neurogenerative processes such as the formation of the blood-brain barrier, myelination, neurogenesis, and microglia maturation” (Sharon et al., 2016, p. 915), then could such microbes also be critical for normal mental functioning? There is now evidence that the microbiome may also be stimulating mammalian social behaviors (Stilling et al., 2018; Sherwin et al., 2019). Germ-free mice have a suit of abnormal behaviors, including excessive time spent in repetitive self-grooming, social avoidance, and very little time spent in social investigation. Desbonnet and colleagues (2014) remarked that these traits appeared to be similar to those of autistic children. Moreover, some of these behavioral traits can be normalized by providing the germ-free mice with gut bacteria early in post-natal life. There are many pathways to accomplish this. Wu and colleagues (2021) have discovered that these “socializing” bacteria (especially *Enterococcus faecalis)* can act, in part, by suppressing corticosterone release from the paraventricular region of the brain. This may make socializing with other mice less stressful. The asocial behavior of germ-free mice may also be due to the deficiency of oxytocin-releasing signals from the vagus nerve. This behavior which can be reversed by providing the germ-free mice with *Lactobacillus reuteri* or with microbes from normal mice or even from neurotypical humans (but not with microbes from some autistic patients; Sgritta et al., 2019; Sharon et al., 2019; Bravo et al., 2011)^8^.

Therefore, the microbiome appears to be critical for normal animal development. In mammals, the gut, pancreas, immune system, and brain form from the interactions of microbial cells with zygote-derived cells. Animals develop sympoietically, in partnership at every level. Indeed, if bacteria help make us into healthy and social animals, both the bacteria and the mammal benefit. Indeed, perhaps we animals are the bacteria’s way of making more environments for our bacteria’s descendants (Gilbert, 2018; 2021).

### 3.4 Reconfiguring biology sympoietically

In contributing to physical and behavioral development, it should not be surprising that microbes are critical to the completion of life-cycles. This can readily be seen in marine invertebrates, where the metamorphosis from larva to adult is often mediated by microbes. In the life cycle of the sponge *Amphimedon*, for instance, symbiotic bacteria are needed to supply the arginine that is utilized to synthesize the nitric oxide cue for settlement of the larvae and their metamorphosis into the adult sponge (Song et al., 2021). Similarly, lipopolysaccharide from the biofilm-forming bacterium *Cellulophaga lytica* is needed for the settlement and subsequent metamorphic events of *Hydroides elegans*, the common tube worm (Freckelton et al., 2022). Recent studies (Freckelton et al., 2024) strongly suggest that the bacteria may be forming a prepattern that would become the template for the biogeographic distribution of bethic marine organisms.

Sympoiesis must be foregrounded as a crucial parameter for the conception of life. First, sympoiesis means the end of our thinking of ourselves and other organisms as monogenomic, self-contained, self-making individuals (Demster, 1998; Gilbert et al., 2015; Haraway, 2016). As Donna Haraway noted, we literally “become one with others.” We receive 22,000 genes from the zygote; we receive over eight million different genes from our symbionts (Funkhauser and Bordenstein, 2013).

If organisms are to be perceived as individuals, we are individual teams. And just like teams, organisms are formed through a mixture of cooperative and competitive processes. One competes to become part of a cooperating team, and “making the team” can be intensely competitive, whether it be for the Chicago Bears or for an ursine holobiont. We saw this in our discussion of *Euprymna* and *Vibrio*. Moreover, like a team, each player forms part of the environment for the other players. The co-symbionts mutually scaffold and provide affordances for each other’s existence (Clark and Gilbert 2022; Chiu and Gilbert, 2020). The symbionts in the rumen allow the existence and propagation of cows; the cows allow the existence and propagation of their ruminal bacteria. There is no “host” in the symbiotic relationship (Haraway, 2016). Animals constitute the environment of bacteria, as bacteria constitute the environment of the animals (Formosinho et al., 2022.)

This has important consequences for evolution. As in sports teams, what gets selected in evolution may be the “team” of organisms, not the individual players (Roughgarden et al., 2017; Osmanovic et al., 2018; Roughgarden, 2020). Evolutionary speciation often occurs, as Lynn Margulis and Dorian Sagan (2002) announced, through “acquiring genomes.” On the microevolutionary level, new combinations of symbionts have given rise to new phenotypes. Whether a pea aphid is green or red, thermotolerant or thermolabile, immune or susceptible to parasitoid wasp infection, depends on the bacteria associated sympoietically with the developing aphid body (Dunbar et al., 2007; Oliver et al., 2009; Tsushida et al., 2010). Similarly, new phenotypes can arise from new combinations of symbionts. The acquisition of new fungal symbionts has enabled the relatively benign red turpentine beetle of the Oregon and Washington states to become a major killer of Chinese pine trees (Sun et al., 2013; Taerum et al., 2013). The acquisition of pesticide-resistant bacteria has allowed the *Riptortus* bean bug to acquire the resistance to insecticides (Kikuchi et al., 2012; Kim et al., 2016; Lee et al., 2019). And, of course, the acquisition of symbionts allowed animals to digest plants.

These modules can be tightly connected (as in *Buchnera’s* being an obligate symbiont of pea aphids; Monnin et al., 2020; Bennett and Moran, 2015) or loosely tied to an animal’s development (as in the above-mentioned cases for a pea aphid’s color, thermotolerance, and parasitoid resistance). In loosely coupled symbioses, the short-lived symbionts can provide adaptation to changing environments (as in metamorphosis). In tightly coupled symbioses (as in Buchnera and pea aphids), the symbiont’s contribution becomes canalized into the normal developmental trajectory (Bennett and Moran, 2015). As Žukauskaitė (2020) points out, “sympoietic systems carry different bits of information in their components … This makes sympoietic systems more flexible and adaptive, in the sense that they can easily adapt to changing environments, and also create something new, produce new forms of organization (in this regard they are allopoietic).” Thus, symbionts add a new modular dimension to evolution. We have shown here that symbionts are critical for normal development. If, as evolutionary developmental biologists insist, evolution is made possible by changes in development, then changes in symbionts can cause changes in evolution.

#### 3.4.1 The warp and woof of biodiversity

Sympoiesis may be the defining characteristic of biodiversity both temporally (as studied by evolutionary biology) and spatially (as studied by ecology). First, in the temporal dimension, major evolutionary innovations may have emerged through new sympoietic associations. This may include multicellularity (Dayel et al., 2011), meiosis (Woznica et al., 2017), herbivory (Gilbert, 2020), and the mammalian uterus (Emera and Wagner, 2012). Each appears to have arisen through new combinations of animals and symbiotic microbes (Gilbert, 2019). Even the animal nervous system may have evolved partly out of a need to orchestrate the complex interactions between animals and their associated micro-organisms (Augustin et al., 2017; Klimovich and Bosch, 2018).

Sympoiesis also seems to be critical in forming the great network that hold the planet’s biodiversity together. The root nodules on legumes, which provide fixed nitrogen into the soil, are neither organs of the plant nor the bacteria. They are made from both participants, and they result from an intimate communication between the plant root and the rhizobium bacteria involving flavonoids and nodulation factors. Similar factors are required to form the symbiosis between the plant roots and the fungi that produce the mycorrhizae. The tidal and coral ecosystems are similarly sustained by sympoiesis. The worldwide coastal tidal zone, is sustained by a quadruple symbiosis involving seagrass, nitrogen-fixing bacteria, clams, and the sulfide-oxidizing bacteria living inside the clam’s gills (Cardini et al., 2022). For the formation of coral reefs, mutualistic unicellular algae (dinoflagellates of the family *Symbiodinaceae*) enter into and reside in the gastric cells of the corals, where they transport up to 95% of their photosynthetically produced carbon compounds to their hosts (Muscatine et al., 1984). The photosynthesis by the algal symbionts fuels coral growth and the calcification that creates the reef. Most coral-algae symbioses are established horizontally, with the larval or post-settlement stage acquiring the algae from the environment. It appears that these early stages of development can acquire different species of algae, and that the interactions eventually favor a single species (del Gomez-Cabrera et al., 2008; Matsuda et al., 2022). Therefore, sympoiesis is crucial in creating the major ecosystems of the planet.

When we talk about life on earth, we are talking about holobionts. Therefore, when we discuss development, we must discuss the development of holobionts. This entails studying the ways that microbes signal developmental phenomena to occur. It also involves studying how these interactions allow the persistence of microbial communities. And when we look at evo-devo, we are studying holobiont evo-devo. The study of life is the study of holobionts and the development of relationships between organisms.

## Data Availability

The original contributions presented in the study are included in the article/Supplementary Material, further inquiries can be directed to the corresponding author.

## Footnotes

1 As we will see, Haraway (2016) has pointed out that the host is also in a symbiotic relationship and is therefore another symbiont. It is just larger.

2 Some animals have been reported to lack symbiotic partners. While rare, these would not be unexpected. In many instances, horizontal gene transfer has given animals the genes that had made their symbionts essential. Two groups of beetles, for instance, have acquired fungal genes whose protein products digest plant cell walls (Kirsch et al., 2014). In at least one instance of an animal devoid of internal symbionts, external bacteria are critical in propelling the organism from the larval to adult stages of its life cycle (Vijayan et al., 2018; Freckelton et al., 2022).

3
*Sympoiesis* is a concept in developmental biology referring to developmental inputs from several sources. We are not merely the products of the zygotic cells; we are created “together” with the help of other species. This has been confused with the notion of *symbiogenesis*, which is a concept in evolutionary biology meaning that evolutionary events such as speciation and the formation of the first cells arose through the acquisition of new genomes (rather than by the mutation of existing ones).

4 When the organism evolves such that something in its environment can now be utilized, the newly perceived item is sometimes called an “affordance.” since the environment *affords* a different opportunity for exploitation (Walsh, 2015).

5 This could be the cell surfaces of the “host” or the community of other microbes that support the membership of a bacterium within a biofilm. Blaser and Kirshner (2007) propose that cross-signaling relationships between humans and their microbes constitute evolutionary stable strategies for holobiont development.

6 This was also seen in the above-mentioned studies analyzing the induction of angiogenin-4 by bacteria. Several bacteria were able to induce angiogenin-4 gene expression; but *Bacteroides thetaiotamicron* did it better than the other species.

7
Gilbert and Tauber (2016) have proposed that the boundary of the holobiont is the immune system, a system created through the interactions of host and symbionts. This system defines “self” in a dynamic manner, changing constantly as new are encountered. “Whatever these interactions are between microbe and host, the holobiont is being continuously constructed.”

8 Although human autism spectrum anomalies probably have many causes, pilot studies have shown that replacing the gut bacteria of severely autistic children with those of neurotypical children can dramatically enhance the sociability of many patients (Kang et al., 2019; Puricelli et al., 2022). A recent international study (Morton et al., 2023) claims to find a set of bacteria and bacterial products in autistic patients that correlates with human brain gene expression changes and pro-inflammatory cytokine profiles.
